# Enhancement and evaluation of a prescription audit system for direct oral anticoagulants using a check sheet

**DOI:** 10.1186/s40780-021-00205-y

**Published:** 2021-06-01

**Authors:** Naoto Ishikawa, Hanae Oshikiri, Shinya Takasaki, Masafumi Kikuchi, Taku Obara, Kazutoshi Akasaka, Masaki Matsuura, Hiroaki Yamaguchi, Nariyasu Mano

**Affiliations:** Department of Pharmaceutical Sciences, Tohoku University Hospital, 1-1 Seiryo-machi, Aoba-ku, Sendai, Miyagi Japan

**Keywords:** Direct oral anticoagulant, Anticoagulant, Prescription, Pharmacist, Intervention

## Abstract

**Background:**

Renal function and use of concomitant medications should be carefully monitored in patients subjected to treatment with direct oral anticoagulants (DOACs); the dose should be individually designed for each patient. Owing to the complex therapeutic indications and dose reduction criteria, pharmacists exercise caution when determining the optimal dose for each patient. A DOAC check sheet has been developed that is automatically printed in the dispensing room at the same time as the prescription and can be used by pharmacists to dispense DOACs promptly and correctly. The purpose of this study was to evaluate the system for dispensing DOACs using a check sheet.

**Methods:**

The study was conducted at Tohoku University Hospital in Japan; prescriptions containing DOACs dispensed by the hospital pharmacists were evaluated. The DOAC check sheet described indications, dosage regimens, dose reduction criteria, and contraindications for each drug and included the patient’s information. The check sheet was set to print automatically in the dispensing room at the same time as the prescription when an inpatient was prescribed DOACs. This check sheet was evaluated using a prescription survey and a questionnaire for pharmacists.

**Results:**

The usefulness of this check sheet for the correct use of DOACs was evaluated. There were four inquiries out of 642 (0.6%) prescriptions from pharmacists to physicians regarding DOAC prescriptions, such as the dose introduced before DOAC check sheet utilization, and there were 21 out of 905 (2.3%) prescriptions when the DOAC check sheet was used it, showing a significant increase (*p* = 0.0089). After the introduction of this sheet, overdoses of DOACs were identified at the time of dispensing. Of the 52 pharmacists who responded to the questionnaire, 51 (98%) stated that the check sheet was useful.

**Conclusion:**

The use of the DOAC check sheet is likely to render safety to DOAC drug therapy for individual patients.

## Background

Direct oral anticoagulants (DOACs) have been used as an alternative to warfarin [1–5]. In Japan, four DOACs, dabigatran, rivaroxaban, apixaban, and edoxaban, are available [4–6]. DOACs are effective against various types of thrombosis [1–5] and are used for the prevention and treatment of stroke and systemic embolism in patients with non-valvular atrial fibrillation (NVAF). Rivaroxaban, apixaban, and edoxaban are effective in treating and preventing the recurrence of venous thromboembolism (VTE), such as deep vein thrombosis (DVT) and pulmonary embolisms (PEs). Edoxaban also exerts a preventive effect on VTE in patients undergoing lower limb orthopaedic surgery (LLOS). DOACs exhibits several advantages over warfarin [5, 6]. DOACs are subjected to dose reduction according to the patient profile, but unlike warfarin, it does not require frequent blood tests, such as for the prothrombin time-international normalized ratio (PT-INR). Additionally, DOAC is less affected by food and concomitant medications, its effects appear quickly after administration, and there is less risk of intracerebral haemorrhage. Because the blood concentration of DOACs fluctuates greatly depending on renal function and concomitant medications [2–5], the risk of bleeding increases due to their high efficacy [7]. On the other hand, real-world surveys have reported that the dose of DOAC was underdose [7, 8]. Therefore, an overdose of DOAC increases the risk of adverse events, and underdose diminishes its effectiveness [7]. That is, it is very important to adjust the dose of DOAC for each patient.

**Fig. 1:**
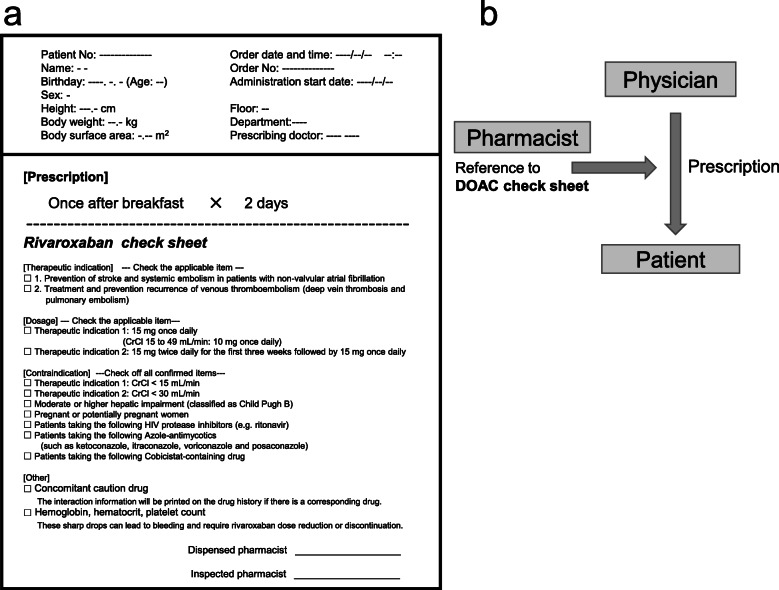
DOAC check sheet (**a**) and dispensing flow for its utilization (**b**)

Because of the complex therapeutic indication and dose reduction criteria for these four DOACs, pharmacists need to take care and time to determine the optimal dose for each patient. Therefore, a DOAC checklist was developed in our hospital. The novelty of this system is that when a physician orders a prescription containing DOAC, it is automatically printed in the dispensing room at the same time as the prescription and can be used for pharmacist dispensing in a timely manner (Fig. 1). This study aimed to investigate the usefulness of the DOAC check sheet.

## Methods

### DOAC check sheet

The DOAC check sheet describes indications, dosage regimens, dose reduction criteria, and contraindications for each drug [2–6], and patient information (age and weight) from the medical support system at this hospital, which is shown in Fig. 1a. The dispensing flow using this check sheet is shown in Fig. 1b. The check sheet was set to print automatically in the dispensing room at the same time as a DOAC was prescribed to the patient. The pharmacist considered the provision of drugs for drug therapy using the check sheet in addition to the prescription and medical information. When necessary, the pharmacist discussed with the physician and dispensed the DOAC as required.

### Evaluation of DOAC check sheet

#### Study 1: number and content of inquiries regarding DOAC prescriptions

Introduction of this check sheet spanned across a period of 3 months, from December 2016 to February 2017, and results were compared with those in the period before the introduction, from August 2016 to October 2016. The comparison items included the number of DOAC prescriptions before and after the introduction of the check sheet, the number of inquiries, and the content of inquiries. To compare the number of inquiries for each drug before and after introducing the check sheet, a chi-squared test was used; a risk rate of less than 5% was considered statistically significant. The number of DOAC overdose prescriptions for the selected patient profiles and pharmacist interventions were investigated.

#### Study 2: questionnaire for pharmacists regarding DOAC check sheets

After the end of the DOAC check-sheet introduction period, a questionnaire survey was conducted with our hospital pharmacists that used the system to evaluate the convenience and effectiveness of the check sheet. The questionnaire included the following questions:

A. Was the DOAC check sheet useful?
B. Which content was useful?
C. Was the DOAC check sheet necessary?
D. What did the DOAC check sheet help you with?
E. What type of awareness did you have with the introduction of the DOAC check sheet?

## Results

### Evaluation of DOAC check sheet

#### Study 1: number and content of inquiries regarding DOAC prescriptions

**Fig. 2:**
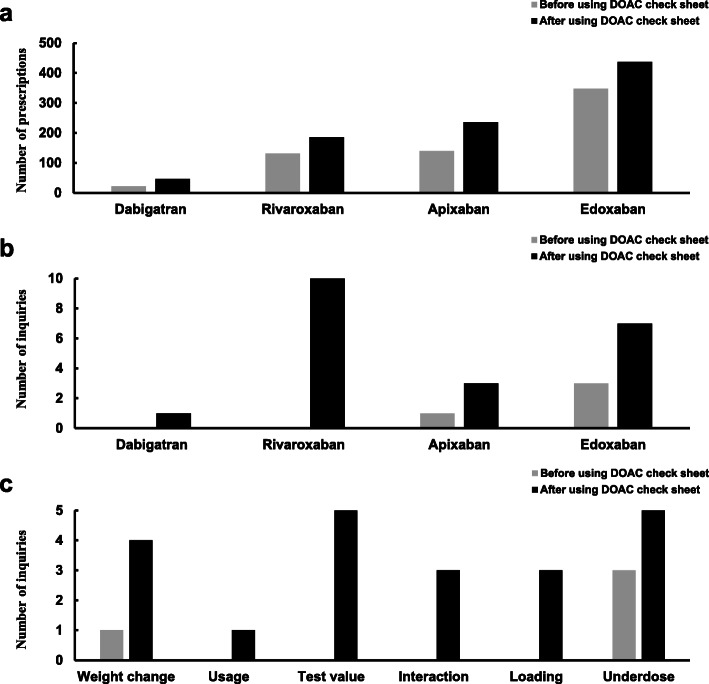
Changes in the number of prescriptions (**a**) and inquiries (**b**) before and after using the DOAC check sheet and their contents (**c**). footnote: **c** was retrospectively classified into weight change, usage, test values, interaction, loading, and underdose based on the inquiry contents

The total number of DOAC prescriptions was 642 before the check sheet introduction and 905 after its use in this study. The results for each drug have been shown in Fig. 2a; the numbers of prescriptions before its use were: edoxaban, 348; apixaban, 140; rivaroxaban, 132; and dabigatran, 22; the results and after its use were: edoxaban, 437; apixaban, 236, rivaroxaban, 186, and dabigatran, 46. About half of the prescriptions were fore edoxaban: apixaban and rivaroxaban were prescribed for approximately 20 to 30% of the prescriptions, and dabigatran prescriptions were less than 10%; the prescription ratio was the same before and after the check sheet use. The number of inquiries from pharmacists to physicians regarding DOAC prescriptions was 4 out of 642 (0.6%) before using the DOAC check sheet and was 21 out of 905 (2.3%) after its use, showing a significant increase (*p* = 0.0089); the increase involved the prescription of all DOACs (Fig. 2b). The questionnaire focused on data acquisition based on weight change, usage, test value, drug interaction, loading, and underdose. When compared before and after the use of the DOAC check sheet, all values increased (Fig. 2c). The details of inquiries and prescription changes for each drug before and after the introduction of the DOAC check sheet are shown in Table 1. The number of DOAC prescriptions for which the dose was subjected to reduction as per the patient’s profile was four before the introduction of the DOAC check sheet and 12 after the introduction. Of these, one out of four prescriptions and 12 out of 12 prescriptions were introduced at appropriate doses with the intervention of the dispensing pharmacist (incidentally, three out of four prescriptions that were overlooked before the introduction of the DOAC check sheet were intervened by the ward pharmacist and were adjusted to the appropriate dose). The introduction of the DOAC check sheet resulted in, no overdose prescriptions being dispensed.

**Table 1:** Inquiry contents and prescription changes before and after the use of DOAC check sheet

	Drug	Number of inquiries	Prescription change	
			Change	No change
**Before using the DOAC check sheet**				
Underdose	Dabigatran	0	0	0
	Rivaroxaban	0	0	0
	Apixaban	1	0	1
	Edoxaban	2	0	2
Overdose	Dabigatran	0	0	0
	Rivaroxaban	0	0	0
	Apixaban	0	0	0
	Edoxaban	1	0	1
**After using the DOAC check sheet**				
Underdose	Dabigatran	1	0	1
	Rivaroxaban	1	0	1
	Apixaban	2	0	2
	Edoxaban	2	0	2
Overdose	Dabigatran	0	0	0
	Rivaroxaban	7	1	6
	Apixaban	1	0	1
	Edoxaban	4	2	2
Other	Dabigatran	0	0	0
	Rivaroxaban	2	1	1
	Apixaban	0	0	0
	Edoxaban	1	0	1

#### Study 2: questionnaire

The questionnaire survey evaluated the utility of the DOAC check sheet among dispensing pharmacists. The response rate was 78% (52 out of 67). 51 (98%) pharmacists stated that the check sheet was useful (Table 2A), particularly, the information (in descending order) on dosage, contraindications, therapeutic indications, concomitant drug cautions, and laboratory test values (Table 2B). The need of this check sheet was assessed by 38 (73%) responders as necessary; 12 (23%) responders assessed the utility as neither necessary nor unnecessary, and 2 (4%) responders assessed it as not unnecessary (Table 2C). The usefulness of this check sheet was considered as “improving the quality of prescription inspections” by 31 (60%) responders, “standardization of prescription inspection” by 26 (50%) responders, “new pharmacist education” by 16 (31%) responders, “improving dispensing efficiency” by 7 (13%) responders, and “ward pharmacist work” by 1 (2%) responder (Table 2D). The awareness after the introduction of the check sheet (Table 2E) was classified as “appropriate confirmation of dosage” by 43 (83%) responders, “frequency of checking drug package inserts” by 37 (71%) responders, “attention to drug interactions” by 20 (38%) responders, “understanding the purpose of prescription” by 9 (17%) responders, “no change” by 3 (6%) responders, and “other” by 1 (2%) responder.

**Table 2:** Questionnaire to pharmacists for evaluating the use of DOAC check sheets

Questionnaire contents to pharmacists (***n*** = 52)	n (%)
**A. Is the DOAC check sheet useful?**	
Yes	51 (98)
No	1 (2)
**B. Which content is useful?**	
Therapeutic indication	24 (46)
Dosage	43 (83)
Contraindication	25 (48)
Concomitant drug caution	22 (42)
Laboratory test value	13 (25)
**C. Do you need the DOAC check sheet?**	
Necessary	38 (73)
Neither	12 (23)
Unnecessary	14 (4)
**D. What did the DOAC check sheet help you with?**	
Improvement in quality of prescription inspections	31 (60)
Standardization of prescription inspection	26 (50)
New pharmacist education	16 (31)
Improvement in dispensing efficiency	7 (13)
Ward pharmacist work	1 (2)
**E. What kind of awareness did you have with the introduction of the DOAC check sheet?**	
Appropriate confirmation of dosage	43 (83)
Frequency of checking drug package inserts	37 (71)
Attention to drug interactions	20 (38)
Understanding the purpose of prescription	9 (17)
No change	3 (6)
Other	1 (2)

## Discussion

The purpose of this study was to enable the proper use of DOACs by constructing a system that automatically provides the check sheet printed with test values and concomitant medications related to the drug dose criteria when DOACs are prescribed (Fig. 1). Dispensing using such a DOAC check sheet is not common; its use, can lead safer DOAC drug therapy. The novelty of the dispensing system that uses the DOAC check sheet is that patient information is automatically printed promptly during dispensing. The total number of DOAC prescriptions before and after the introduction of the check sheet was different, but the ratio of the drugs used was the same before and after the introduction, with edoxaban, apixaban, rivaroxaban, and dabigatran being used in descending order of prescription (Fig. 2a). Edoxaban dose can be easily modified to set because it is the same for NVAF and VTE, and it is the only DOAC indicated for the prevention of VTE in patients with LLOS. These are likely the reason for the high prescription rate. Dabigatran has a neutralizing effect on coagulation [9, 10], is less affected by CYP3A4 metabolism than other DOACs, and its coagulation effect can be predicted by test values such as activated partial thromboplastin time [11, 12], thereby rendering it safer for use in patients with multiple drugs; however, in this study, its prescription rate was less than 10%, possibly because it is the only indication for venous thrombosis. The number of prescription inquiries increased significantly from 4 before to 21 after the check sheet introduction (Fig. 2b), all of their contents also increased (Fig. 2c). It is presumed that this increase in the number of questions relating to all drugs was due to the dispensing pharmacists being provided with accurate and reliable information on the dose-reduction criteria for each of the four DOAC drugs by using the check sheet. For the subsequent content inquiry, the check sheet was well organized in terms of contraindications, interactions, and test values that invited additional questions from pharmacists. The introduction of this check sheet at the time of dispensing eliminated overdosing by DOACs that has been reported to cause the occurrence of serious adverse events (Table 1). In Japan, ward pharmacists are rarely stationed on holidays and at night, this check sheet can be used to ensure appropriate dosing with DOACs at any time of the day.

Evaluation of the DOAC check sheet using the questionnaire showed (Table 2) that, 98% of the respondents considered was useful for the purpose. Many responders believed that it helped to improve and standardize the prescription inspections, but 13% of the responders considered the efficiency of dispensing to be low, mainly because the indications for DOACs cannot be confirmed by prescription alone. It was necessary to refer to the electronic medical record to confirm the patient’s indication; thus, it was difficult to improve the dispensing efficiency with the utilization of this sheet alone. In the future, it will be necessary to develop a system that would confirm the patient’s indications using the prescription at the time of dispensing.

This study has the following limitations: the study period was short and the study was conducted at a single facility. In the future, a similar system should be constructed at multiple facilities and should be subjected to evaluation over a longer period. In Japan, the number of facilities that print clinical test values on prescriptions has increased recently; however, the utility of these data depends on the extent of pharmacists’ knowledge and experience. By using a check sheet that describes not only the test values but also the information necessary for dose evaluation like the system presented here, the quality of all examinations is improved regardless of the individual pharmacist’s skills. We expect that such improved systems will be used to other drugs and will contribute to safe and effective drug therapies.

## Conclusion

The DOAC check sheet was prepared and the system was constructed to print it at the same time as the prescription to assist in the correct use of DOACs. Its use resulted in an increased number of appropriate drug- and indication-related inquiries by pharmacists during dispensing and helped to prevent events of DOAC overdosing. The usefulness of this system was also confirmed through responses to a questionnaire by pharmacists who used it. Therefore, this system can contribute to the safety of drug therapy for individual patients treated with DOACs.

## Data Availability

All data generated or analyzed during the study are included in this published article.

## Abbreviations

CYP: Cytochrome P450
DOAC: Direct oral anticoagulant
DVT: Deep vein thrombosis
GIH: Gastrointestinal haemorrhage
LLOS: Lower limb orthopaedic surgery
NVAF: Non-valvular atrial fibrillation
PE: Pulmonary embolism
PT-INR: Prothrombin time-international normalized ratio
VTE: Venous thromboembolism
